# Current Hospital Topics

**Published:** 1906-07-21

**Authors:** 


### HOSPITAL ADMINISTRATION.

### CONSTRUCTION AND ECONOMICS.

### CURRENT HOSPITAL TOPICS.

**A Strong Indictment against Present Free Medical Relief.**

Doctor Donald F. Shearer insists in the *Times* that the national character and moral must suffer disaster from the fact that two-thirds of the population of London are habitual recipients of charity from the hospitals and other institutions maintained by the rates or by voluntary subscriptions and endowments. He goes on to show that so great is the proportion of Londoners who habitually use the hospitals that the death-rate per thousand in London as represented by institution mortality is 5.2 per cent., whereas it is only 1.3 per cent. in the whole of the rest of England and Wales. It appears that 41 per cent. of the deaths, apart from infants, in London, as against 15 per cent. in the rest of England, are deaths of persons in the actual receipt of charitable or gratuitous medical relief. Dr. Shearer insists that it is no exaggeration to say that two-thirds of the persons who die in London, infants excepted, have been treated gratuitously in hospitals for the diseases of which they die. " The out-patient and casualty departments in the London hospitals are a disgrace to any community." Including St. Bartholomew's and St. Thomas's Hospitals the records show seven million attendances each year at the institutions supported by the Sunday Fund. He further maintains that at any one time the number of persons under free medical treatment in London is over 200,000, and that at least three millions of the population of London receive free medical treatment from time to time, and do not in any way consider it a degradation to receive such charity. Dr. Shearer concludes his indictment: " The popular clamour for *panem et circenses* heralded the fall of Rome: our modern equivalent is free education, free medicine, free meals, free pensions, and, last of all, free spectacles! God forbid that this craze for giving and getting, encouraged though it be by the highest in the land, should lead to a like ending." There can be no doubt, as we have so often contended, that the amount of free medical relief at present given by the hospitals in London is hugely greater than the needs of the poorer population who are alone entitled to receive it. Why do not the hospitals, or some of the more intelligently managed of them—say one, at least, to show the way—display the courage to cut down their expenditure by reducing the amount of free relief and strictly confining it to those alone whose circumstances justify the gift?

**Mr. A. E. Reade.**

Mr. Reade has retired from the post of secretary to Charing Cross Hospital, which he has held since 1882. Mr. Reade was born at Benares on March 8, 1848; he was the youngest son of Mr. E. A. Reade, C.B., a Bengal civilian, who distinguished himself in the Mutiny, and a nephew of the late Charles Reade the author and dramatist. Mr. Reade was educated at Haileybury, and from 1868 to 1879 was assistant superintendent of Indian Government telegraphs. In 1880 he was appointed secretary to the Hospital for the Paralysed and Epileptic, from which hospital he migrated to Charing Cross on his appointment as secretary to that institution. When Mr. Reade commenced his duties Charing Cross Hospital was a relatively small and unimportant hospital very much in need of reorganisation and reconstruction. There was no operation theatre as such, and an old resident states that " the patients were anæsthetised on the table, a barbarous proceeding with male patients, and shocking in addition in the case of females." We propose to deal at length with the developments and additions to Charing Cross Hospital on another occasion, but it is bare justice to state that during Mr. Reade's period of office the site has been materially added to, proper accommodation has been provided for the resident staff, a new nurses' home has been built, the hospital itself has been much enlarged and reconstructed throughout, the medical school has been enlarged and completely equipped, new operation theatres with a clinical laboratory and an *x*-ray department, new out-patient and casualty departments, new surgical wards, four isolation wards, an electrical department with baths, and new kitchens and heating apparatus have been provided, the total cost, including the site, being upwards of £170,000. This expenditure has entailed the sale of practically the whole of the invested funds of the hospital, and the problem at the moment is to pay off the heavy sums owing to managers and others, and to provide the institution with an adequate income. Mr. Reade retired, we understand, upon a pension, and will continue to do some work still under the direction of the treasurer. Mr. Reade has a host of friends who will welcome his retirement, because it will enable them to see more of their friend whose genial personality is gratefully welcomed on all occasions.

**The League of Mercy.**

The principle on which this League has been successfully developed, until it has to-day attained its present widespread and ever-increasing influence, is that of personal service in the days of health in the cause of the sick. The number of presidents and lady presidents working under T.R.H. the Prince and Princess of Wales is 162, with whom are associated some 1,200 ladies and gentlemen acting as vice-presidents. These directly guide the work of some 20,000 members, whose business it is to enrol as associates those who are willing to give one shilling a year or upwards to the League. The large and increasing number of persons thus associated with the League is undoubtedly due to the fact that all know what they give is handed over to the hospitals without any deduction whatever, the expenses of the League being met by the subscriptions of honorary members who are unable to render personal service. The garden party at Marlborough House on the 12th instant was probably the most successful and charming entertainment of the season. This garden party is given annually by the Grand President and Lady Grand President of the League, in order that they may receive as guests in due turn every worker connected with the League who renders continuous personal service in the cause of the sick and suffering. The example set by the Prince and Princess of Wales in giving this garden party to workers for the League is followed by the Presidents and Vice-Presidents, who ask to a similar function the vice-presidents and members in their divisions, so that they may have an opportunity of seeing all the workers in their district and that the interest in the work may be sustained and extended. In the same way the vice-presidents assemble their members and entertain them, so that, apart from the direct benefit which the League has conferred by contributing upwards of £60,000 to the London hospitals, it has exercised an immense influence for good over tens of thousands of people, who are cheered by their connection with this organisation, and many of whom have acknowledged the happiness and benefit which they have individually received by joining in the work. All who were present at Marlborough House on the 12th instant must recognise that the Prince and Princess are deliberately rendering a considerable measure of personal service themselves for, apart from other work of an important character which they each do for the League every year, it is no small service to spend three hours in an endeavour to speak personally to every one of the workers for the sick who are present at the garden party each year. It would be difficult to over-estimate how much these garden parties have done for the success of the League of Mercy. It is properly felt to be an honour to be received as a guest at Marlborough House, and T.R.H. have exceeded the traditional limits of rank and station by welcoming to their home all ranks of society, from the highest to the humblest, who have shown themselves eager to serve the cause of the sick and suffering by becoming associated with the League of Mercy.

